# Impact of Culinary Treatments on the Immunoreactivity of Soy Protein Isolates

**DOI:** 10.3390/foods15010001

**Published:** 2025-12-19

**Authors:** Anna Jędrusek-Golińska, Dorota Piasecka-Kwiatkowska, Krystyna Szymandera-Buszka, Marzanna Protasiewicz

**Affiliations:** 1 Department of Gastronomy Science and Functional Food, Poznań University of Life Sciences, Wojska Polskiego 28, 60-637 Poznań, Poland; anna.jedrusek-golinska@up.poznan.pl (A.J.-G.); krystyna.szymandera-buszka@up.poznan.pl (K.S.-B.); marzanna.protasiewicz@up.poznan.pl (M.P.); 2 Department of Food Biochemistry and Analysis, Poznań University of Life Sciences, Mazowiecka 48, 60-623 Poznań, Poland

**Keywords:** soybean isolates, boiling, pressure cooking, microwave cooking, antigenicity

## Abstract

The reduction in the immunoreactive properties of soy proteins is crucial, considering the widespread use of soy in food, including protein isolates. This study aimed to evaluate the effects of various culinary treatments on the immunoreactivity of whole soybeans and isolated 7S and 11S protein fractions. Soybean and the isolated 7S and 11S fractions were subjected to boiling (100 °C for 60 and 120 min), pressure cooking (120 °C, 202 kPa for 50 min), and microwave heating (360 W for 30 min). The immunoreactivity of the samples was assessed using ELISA and Western blotting. Culinary treatment of whole soybeans, regardless of the method, decreased the content of immunoreactive 7S and 11S fractions by approximately 30%. Culinary processing of the isolated 7S and 11S proteins, in the absence of the protective seed matrix, resulted in a more pronounced reduction in immunoreactivity. Pressure-cooking allowed for the reduction of the content of immunoreactive 7S and 11S proteins by 71 and 58%, respectively. Western blotting confirms a decrease in both 7S and 11S immunoreactive bands, with a more marked reduction observed for the 11S fraction. These findings indicate that such a reduction may be sufficient to lower the risk of allergic reactions in individuals with mild soy allergy. However, the persistence of immunoreactive bands even after intensive treatment suggests that culinary methods alone are unlikely to ensure safety for highly sensitized individuals.

## 1. Introduction

Soybeans are a valuable raw material widely used in the food industry due to their high nutritional value. They serve as a common vegetarian and vegan alternative to meats, a substitute for cow’s milk and dairy products, particularly for people with lactose intolerance, and are also used in the production of oil and fermented foods such as miso, tempeh, tofu, and soy sauce. The health benefits of soy consumption are well documented and include a reduced risk of type 2 diabetes, cardiovascular diseases, and osteoporosis [1,2,3].

Soybeans are widely used in the food industry, also as a functional ingredient in the production of a wide range of products [4]. This widespread application is largely attributed to the versatile properties of soy proteins, which are utilized in the formulation of meat and dairy alternatives. These properties include solubility, water-binding capacity, swelling, viscosity, gelling ability, and surface activity. Moreover, soy protein ingredients can be stored in either liquid or solid form (e.g., powder, concentrate), offering flexibility in product formulation and shelf-life management [5,6,7,8,9,10,11,12,13].

Along with milk proteins, peanuts, eggs, fish, and cereals, soy is one of the Big Eight food allergens [8,9]. However, the prevalence of allergy to these major allergens varies considerably by continent and country. In the USA and other developed countries, soy has been considered one of the major food allergens [7], with soy allergy affecting 0.4–0.8% of the population, depending on the age group [13]. A study conducted in China showed that patients with soy allergy accounted for 3% of all food allergic individuals [12]. According to Spolidoro et al. [10], the reported prevalence of soy allergy in Europe is lower than for any of the other seven major allergens. Although some studies suggest that soy can be removed from the Big 8 list without risk to public health [11], a proportion of the population remains hypersensitive to soy protein. This is supported by data from Spolidoro et al. [10], which indicate that, with few exceptions, the prevalence of food allergy and soy sensitization in Europe has not changed significantly over the past decade. The overall self-reported prevalence of soy allergy across all age groups in this continent was estimated at 0.5% (95% confidence interval 0.3–0.7), while the prevalence confirmed by an oral food challenge test was 0.3% (95% confidence interval 0.1–0.4), with a higher prevalence in children than in adults.

An immune response can be triggered not only by the consumption of nutritionally valuable soy storage proteins but also by protein preparations derived from soy [14]. Since many processed food products contain soy-derived ingredients, avoiding soy, especially its hidden forms such as isolates, concentrates, or additives, poses a significant challenge both for allergic individuals and for broader food safety efforts [15].

Stevenson et al. [16] identified sixteen soybean allergen sequences with evidence supporting their sensitization and elicitation potential. Ladics et al. [17] confirmed the clinical significance and the sequence of eight of these allergens [4,14,18,19,20,21,22,23,24].

Among soy allergens, the 7S (β-conglycinin) and 11S (glycinin) fractions are considered the most immunoreactive [19,25,26]. De Angelis et al. [27] demonstrated that 11S globulin is highly resistant to proteolysis in the gastrointestinal tract, and both 7S and 11S fractions retain linear epitopes with sequences homologous to those found in other legume species. This raises the risk of cross-reactions in allergic consumers. Bittecourt et al. [26] showed the presence of two classes of antibodies (IgM and IgG) directed to 7S and 11S fractions in mice, confirming their high immunogenic potential. The 7S fraction also showed high allergenicity, including mast cell degranulation and histamine release via IgE-mediated mechanisms.

The risk of allergies can be reduced by processing that modifies the soy protein structure [16,28,29]. Physical treatments, such as heat, ultrasound, high pressure, and radiation, have been shown to reduce the immunoreactivity of soybeans [30,31]. These methods affect the secondary and tertiary structures of soy proteins, which may lead not only to the destruction of conformational epitopes, but also their masking or unintended exposure [8,30]. Biochemical processes, particularly fermentation and enzymatic hydrolysis, appear to be more effective in degrading allergenic epitopes [32]. However, although these processes significantly reduce epitope immunoreactivity, they do not entirely eliminate the allergenic potential of soy proteins [33,34,35]. It is important to note that hydrolysis can lead to the formation of low-molecular-weight peptides containing proline, leucine, tyrosin, and phenylalanine, which are associated with a bitter taste [28,33,35,36,37,38,39].

Soy isolates may trigger allergic reactions more frequently than whole soybeans, partly due to their hidden presence in processed foods [5,40]. Despite being listed on product labels, they are not always recognized by consumers as allergenic ingredients [41].

The aim of the study was to assess the effect of selected, commonly used household cooking methods, such as varying cooking times, heating in a microwave oven, and cooking under pressure, on the immunoreactive properties of soy proteins present in whole soybeans and soy isolates.

## 2. Materials and Methods

### 2.1. Material

The soybean seeds (*Glycine max*, Augusta variety, non-GMO) were purchased commercially in a retail store in Poznań. The seeds came from a crop in Poland (Lublin region), harvested in 2024, batch number 1366/2/5. They were stored in a sealed container at room temperature in the dark.

### 2.2. Methods

**Figure 1:**
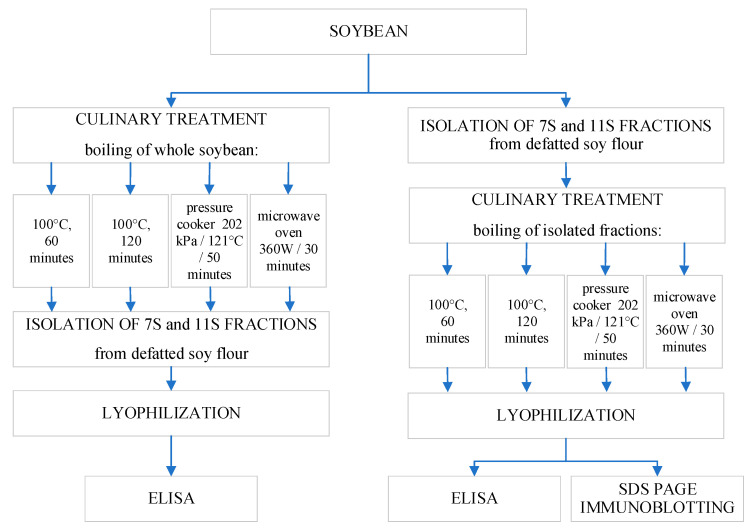
Schematic overview of the study.

The research model is presented in Figure 1.

#### 2.2.1. Isolation of Protein

The process of isolating individual fractions of soy protein consisted of extracting them from defatted soy flour in an alkaline environment, and then in isoelectric precipitation, according to the procedure described by Nagano [42], with modifications proposed by Liu [25]. The following labels were used for the obtained fractions: 7S(s) and 11S(s) for fractions obtained from whole cooked soybean seeds and 7S(i) and 11S(i) for fractions obtained from isolated and cooked soy protein. Culinary treatments were marked as follows: boiling 100 °C, 60 min—b60, boiling 100 °C, 120 min—b120, pressure cooker 202 kPa, 50 min, 121 °C—pc, and microwave oven 360 W, 30 min—mv.

#### 2.2.2. Protein Content

The protein content in soybean seeds and soy isolate was determined by means of the Kjeldahl method [43]. To calculate nitrogen content into protein content, the conversion factor 6.25 was used. Soluble protein was determined by the Bradford method [44].

#### 2.2.3. Protein Extraction from Analyzed Samples

Protein was extracted using 0.1 M TBS-HCl, pH = 8.6, with 2% 2-mercaptoethanol, 2% SDS, and 0.05% Tween 20 additions. This combination of ionic and non-ionic detergents, together with the reducing agent, is widely used in commercial allergen-detection kits in Japan because it efficiently disrupts protein aggregates and matrix interactions. Higher SDS concentration (2%) is particularly effective in releasing matrix-bound soy proteins, markedly improving extraction yield compared to standard buffers. The samples were mixed (room temperature, proportion *w*:*v* 1:10), shaken (1 h), and centrifuged (5000× *g*, 4 °C). The extraction was made in triplicate and then pooled into one sample.

#### 2.2.4. SDS-PAGE Procedure

The electrophoresis was run with the Bio-Rad Mini-PROTAN system (Los Angeles, CA, USA) at a constant temperature of 4 °C, with voltages of 90 V for the stacking gel and 170 V for the resolving gel. The 7S(i)/11S(i) and 7S(s)/11S(s) fractions were analyzed in two independent SDS-PAGE runs. For each run, the amount of protein applied to the gel was adjusted during method optimization to obtain clear and well-resolved protein profiles. Consequently, 6 µg of protein was loaded for the isolated fractions (7S(i), 11S(i)) and 8 µg for the fractions obtained directly from soybean seeds 7S(s), 11S(s). The gels were stained with Coomassie Brillant Blue R-250. The analysis was performed using CLIQS software ver. 1.1. (TotalLab Quant, Newcastle-Upon-Tyne, UK).

#### 2.2.5. Western Blotting Procedure 

After separating with SDS-PAGE, the proteins were transferred to a polyvinylidene difluoride (PVDF) membrane (Immobilone-P, 0.45 μm, Merck Millipore Ltd., Burlington, MA, USA) using the semidry Amersham Biosciences TE 77 PWR system (UK), with a constant current of 0.9 mA/cm^2^. Next, the membranes were blocked for 1 h with 0.01 M TBS, pH 7.4, containing 1% BSA (Sigma A7906, Sigma-Aldrich, St. Louis, MO, USA). Polyclonal anti-soy antibody (Sigma S2519, Sigma-Aldrich, St. Louis, MO, USA) was diluted 1:1000 in blocking TBS 1%-BSA buffer and incubated with protein for 1 h. Then, they were fivefold washed. Next, the membranes were incubated with anti-rabbit IgG (γ-chain specific) mouse monoclonal antibody conjugated to alkaline phosphatase (Sigma A2556, Sigma-Aldrich, St. Louis, MO, USA) diluted 1:8000 with blocking buffer containing 0.05% Tween 20 (Sigma P9416, Sigma-Aldrich, St. Louis, MO, USA) for 1 h. After fivefold washing, the membrane substrate BCIP/NBT (5-bromo-4-chloro-3′-indolyphosphate and nitro blue tetrazolium, Calbiochem, SanDiego, CA, USA) was applied. After 20 min, the reaction was stopped with water. Then the membranes were air-dried in the dark and afterwards analyzed with the CLIQS program software ver. 1.1. (TotalLab Quant, Newcastle-Upon-Tyne, UK) [33].

#### 2.2.6. ELISA Procedure

Soy standards (seeds—7S and 11S fractions) and samples, prior to labeling, were subjected to chemical (urea and mercaptoethanol) and thermal denaturation (100 °C for 1 h), and, before use in the assay, were diluted in 1 mM Tris-HCl buffer, at pH 8.6. Chemical denaturation and sample dilution were performed in accordance with the recommendations of the antibody producer (Sigma S2519). Microtiter plates (Nunc-Immuno™ Micro well Maxisorp™ 96 well polystyrene plates, Thermo Fisher Scientific, Waltham, MA, USA) were coated with antigen (soy standard and samples each in triplicate) and incubated for 2 h at 37 °C. The plates were then washed four times using wash buffer (TBS containing 0.5% Tween 20). Subsequently, the wells were blocked with 200 µL/well of 1% bovine serum albumin in TBST, overnight at 4 °C. Next, the plates were washed four times with wash buffer before adding 100 µL/well of polyclonal anti-soy antibody (Sigma S2519) diluted 1:2500 in TBST-BSA and incubated for 2 h at 37 °C. The plates were then washed four times with 200 µL/well of wash buffer, and anti-rabbit HRP conjugate diluted 1:10,000 in TBST-BSA was added, and the plates were incubated for 1.5 h at 37 °C. After that, the plates were washed four times with the wash buffer before adding 100 µL of OPD (o-Phenylenediamine) substrate to each well. The plates were then incubated for 30 min at room temperature in the dark, and the reaction was stopped by adding 100 µL of 0.16 M sulfuric acid to each well. The optical density (absorbance) of each well was measured using a microplate reader, Asys UVM 340 (Biochrom Ltd., Cambridge, UK) at 490 nm. All measurements were performed in triplicate, and the results were presented as average values. For treated fractions, values were expressed as the percentage of remaining immunoreactive protein relative to the untreated control, which was considered as 100%.

### 2.3. Statistical Analysis

The data were expressed as mean ± standard deviation (SD). The results were analyzed using one-way analysis of variance (ANOVA). When a significant F-value was obtained (*p* < 0.05), differences between means were evaluated using Tukey’s post hoc test. All statistical analyses were conducted using Statistca 13.3 (TIBICO Software Inc., Palo Alto, CA, USA).

## 3. Results

The first stage of the research involved determining the basic chemical composition of raw soybean seeds, as well as the proximate composition of the 7S and 11S protein fractions isolated from both raw and thermally processed seeds. As shown in Table 1, the raw soybean seeds contained 43.30% protein and 20.03% fat on a dry weight basis. As expected, the protein content of the isolates differed from that of the whole soybean seeds, reflecting the protein concentration and purification achieved during the fractionation process. Although slight variations in protein content were observed, no statistically significant differences were found between the 7S and 11S fractions isolated from raw seeds and those obtained after different culinary treatments (Table 1). On average, the 7S and 11S isolates contained 98.16% and 96.25% protein, respectively. Fat was not detected in any of the fractions, regardless of the processing method.

### 3.1. ELISA

The content of immunoreactive proteins was determined by ELISA method in: 7S(s) and 11S(s) fractions obtained from whole soybeans, which were first subjected to culinary processing; as well as in fractions 7S(i) and 11S(i) which were first isolated and then subjected to the same culinary processes as whole soybeans.

For the 7S(s) and 11S(s) fractions, the results indicated that, regardless of the thermal process applied to the soybean seeds, the reduction in immunoreactive protein content ranged from 31.54% to 34.62%, with no statistical differences observed. This relatively modest and uniform response across treatments likely reflects the protective effect of the soybean seed matrix, in which the 7S and 11S globulins are naturally embedded. Thermal processing can induce various modifications of the food matrix, including protein denaturation, aggregation, hydrolysis, disulfide bond rearrangements, and chemical interactions with other components such as carbohydrates and lipids. These reactions may either reduce allergenicity (by masking or destroying epitopes) or increase it (by exposing epitopes or generating new ones). In the present study, considering the specific culinary processing methods applied, the matrix appears to have provided a comparable degree of protection across all treatments. Since no changes in the immunoreactive protein content resulting from various cooking processes were observed, there was no indication for further analyses, i.e., SDS-PAGE and Western blotting.

In contrast, significant changes in immunoreactive protein content were observed in the 7S(i) and 11S(i) fractions subjected to a culinary treatment (Table 2).

The results proved that culinary treatment had a significant impact on the reduction in immunoreactive protein content in both fractions.

In the case of 7S(i) fraction, the most destructive method was pressure cooking, which resulted in the degradation of over 71% of the proteins. In contrast, the least effective method was conventional cooking at 100 °C for 60 min, which reduced the content by only 26%. Cooking time was found to be a critical factor: extending the cooking duration to 120 min led to an additional reduction of 27.4%, resulting in a total loss of 53.85% of the 7S(i) fraction.

The 11S(i) fraction exhibited a different sensitivity to thermal treatment. Microwave heating and cooking at 100 °C for 60 min had the least effect, reducing its immunoreactive protein content by about 38%. However, prolonging the cooking time to 120 min caused a significant reduction, up to 65%.

### 3.2. SDS-PAGE

**Figure 2:**
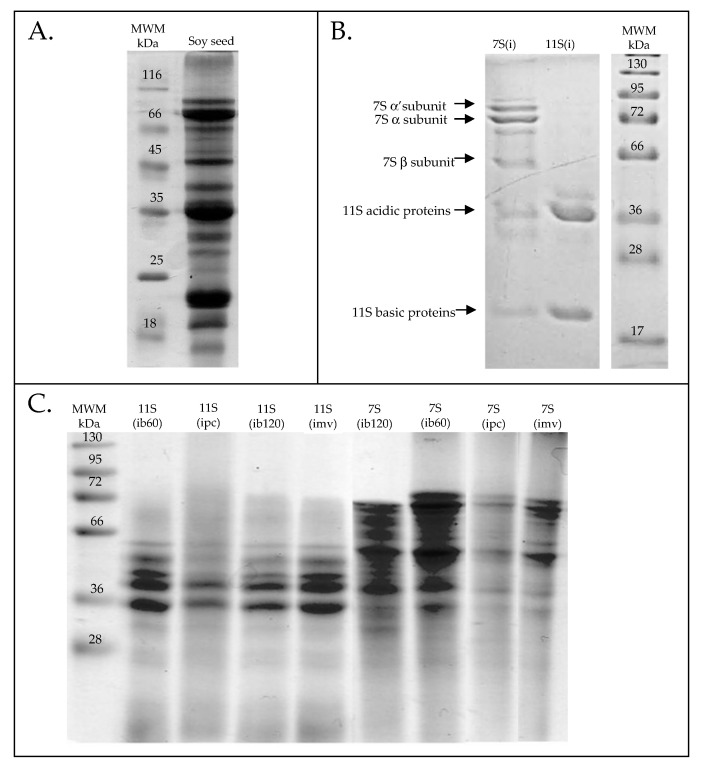
SDS-PAGE analysis of soy seed proteins: (**A**) Whole seeds with molecular weight marker (MWM); (**B**) Isolated 7S and 11S fractions from seeds with MWM; (**C**) MWM with 11S and 7S fractions subjected to different culinary treatments: boiling for 60 min (ib60), boiling for 120 min (ib120), pressure cooking (ipc), and microwaving (imv).

SDS-PAGE electrophoresis was used to characterize the protein profile of raw soybean seeds as well as the 7S(i) and 11S(i) protein fractions isolated from them, including those that were subsequently subjected to thermal processing (Figure 2) [7,8,9,19,39,45,46,47,48,49,50,51,52,53].

Densitometric analysis of the electrophoretic profile of raw soybean seeds (reference sample) revealed protein bands corresponding to molecular weights of approximately 16–18, 20–22, 26–28, 34, 50, 65, and 82 kDa. These values are in close agreement with the molecular weight ranges reported for major soybean seed storage and allergenic proteins in the literature [45,46,47,48,49]. As SDS-PAGE yields approximate molecular weight estimates, minor variations between studies are expected, yet the bands observed here fall within the commonly cited ranges. In the 7S(i) fraction (β-conglycinin) isolated from raw seeds, seven distinct protein bands were detected, with molecular weights ranging from 18 to 90 kDa. According to the literature, β-conglycinin is a protein complex with a total molecular weight of 180–240 kDa, composed of three subunits: α (63.2 kDa), α’ (65.1 kDa), and β (47.9 kDa) [19,50]. In the 7S(i) isolate, two protein bands with molecular weights of 63 and 65 kDa were identified, corresponding to the α and α’ subunits of β-conglycinin, along with a band at 46 kDa corresponding to the β-subunit. Additional protein bands were also observed in the 7S(i) isolate at approximately 20, 35, 80, and 95 kDa. The 20 kDa band likely corresponds to the Kunitz trypsin inhibitor and the 34 kDa band to the P34 serine protease; both are recognized as allergenic proteins [7,8,9,39,51]. The 80 and 95 kDa bands likely represent other minor soybean proteins co-extracted during the isolation process. It should be emphasized that the isolation of 7S(i) and 11S(i) fractions was not intended to obtain high-purity preparations. Rather, in line with the composition of soy protein isolates and concentrates used in the food industry, the aim was to obtain fractions in which the target proteins are predominant, while other minor proteins—including some allergenic ones—may remain present.

In the 11S(i) fraction (glycinin), isolated from soybean seeds, three main protein bands were observed at 20, 39, and 46 kDa, with the first two being dominant. Glycinin is a hexameric storage protein composed of subunits with molecular weights of 53.6, 52.4, 52.2, 61.4, and 66.7 kDa. Each subunit consists of an acidic and basic polypeptide chain [19,52]. According to Ruiz-Henestrosa et al. [53], the 39 kDa band likely corresponds to the acidic chain, while the 20 kDa band represents the basic chain of glycinin. As in the case of the 7S(i) isolate, a less intense additional band at 46 kDa was also detected.

Analysis of the SDS-PAGE profiles of 7S(i) and 11S(i) fractions after thermal treatment revealed that the most pronounced changes occurred following pressure cooking. In the glycinin fraction, only two bands remained visible: those at 39 and 46 kDa. The band corresponding to 20 kDa was no longer detectable, suggesting its degradation. Pressure cooking also caused the most significant alterations in the protein banding pattern of the 7S(i) isolate.

### 3.3. Western Blotting

**Figure 3:**
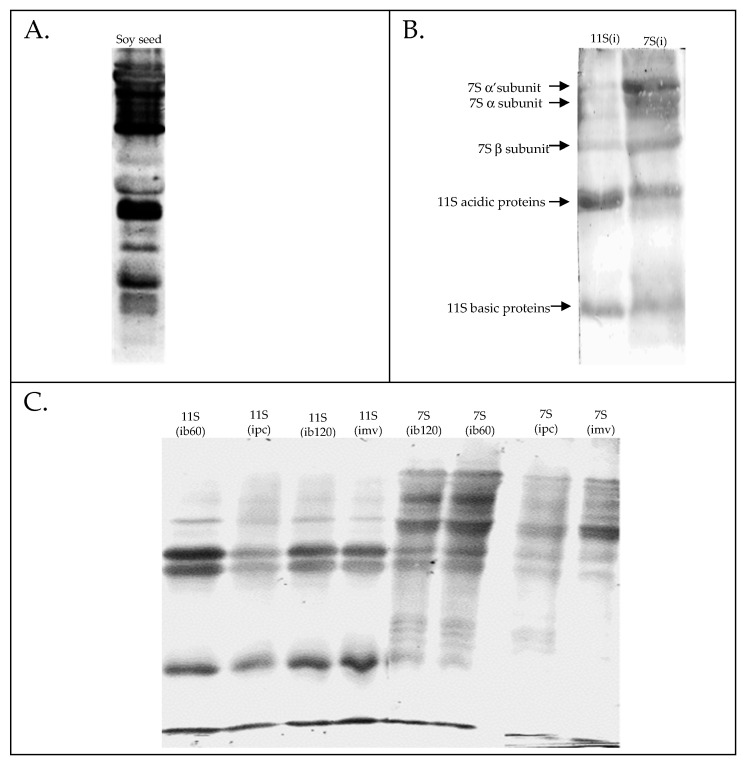
WB analysis of soy proteins: (**A**) Whole seeds; (**B**) isolated 11S and 7S fractions; (**C**) 11S and 7S fractions after culinary treatments: boiling for 60 min (ib60), boiling for 120 min (ib120), pressure cooking (ipc), and microwaving (imv).

Figure 3 presents Western blot membrane images, showing electrophoretically separated proteins from extracts of raw soybean seeds, isolated 7S(i) and 11S(i) fractions, as well as fractions subjected to various culinary treatments. The membrane patterns confirm a high level of immunoreactivity across all tested protein samples. Differences were noted in both the number and apparent intensity of immunoreactive bands depending on the type of treatment applied. The 7S(i) fraction exhibited a stronger reaction with polyclonal anti-soy antibodies, as indicated by the presence of 12 immunoreactive bands compared to 6 bands in the 11S(i) fraction. The greater number of immunoreactive bands supports the conclusion that the 7S(i) fraction has a higher immunoreactive potential, in agreement with previous studies [26].

Thermal processing affected the immunoreactivity of the 11S(i) and 7S(i) fractions to varying degrees. The most intense bands were observed in both fractions after conventional boiling for 60 min, indicating that this treatment was the least effective in reducing immunoreactivity. Densitometric analysis was used to compare the effectiveness of the different culinary treatments, with band intensities expressed as percentages relative to the 60 min boiling samples. Pressure cooking caused the most pronounced reduction in immunoreactivity, decreasing band intensity to approximately 25% in the 11S(i) fraction and to about 30% in the 7S(i) fraction. Boiling for 120 min resulted in only minor additional reductions (by approximately 10%) in either fraction, indicating that prolonged boiling does not substantially diminish immunoreactivity. Microwave treatment had minimal effect on the 11S(i) fraction, while in the 7S(i) fraction, slightly fewer protein bands reacted with immunoglobulins compared to conventional boiling; however, the observed reduction (by approximately 35%) was still less substantial than that achieved with pressure cooking.

## 4. Discussion

We analyzed immunoreactivity of isolated 7S(i) and 11S(i) fractions, with the use of the immunoblotting technique [54,55,56].

Threshold doses in soy-allergic patients vary widely. Subjective symptoms have been reported following cumulative doses as low as 10 mg and up to 50 g of soy protein, whereas objective symptoms have been triggered by doses between 454 mg and 50 g. Importantly, individual dose thresholds did not correlate with symptom severity. Therefore, even a slight reduction in the immunoreactivity of either Gly m 5 or Gly m 6 is of value and worth pursuing, as it may allow individuals with lower sensitivity to safely consume soy-containing products. Our research clearly indicated that culinary treatment can significantly impact the immunoreactivity of 7S(i) and 11S(i) proteins. Similarly, in a study by Villa et al. [57], strong immunoreactivity of soy trypsin inhibitor was reported in model hams, with autoclaving markedly reducing the signal due to protein fragmentation (17–18 kDa) and aggregate formation (25–40 kDa). In their study, the effects of heat treatment and the food matrix on the immunoreactivity of legume proteins (soybean and lupine) were evaluated using SDS-PAGE and immunoblotting. All applied thermal treatments, including baking, mild oven baking, and autoclaving, reduced protein immunoreactivity, with autoclaving being the most effective. In our study, isolated 7S and 11S fractions were subjected to boiling, microwave heating, and pressure cooking. Similar to Villa et al., all thermal treatments decreased immunoreactivity, with pressure cooking showing the strongest effect. Differences between studies likely reflect variations in sample matrices and processing conditions, yet both demonstrate the same overall trend—thermal treatment effectively reduces legume protein immunoreactivity. Bu et al. [58] investigated the effect of combining high hydrostatic pressure (400 MPa) with heat treatment (100 °C) on soy-derived β-conglycinin. They showed that this method of processing significantly reduced the antigenicity and immunogenicity of β-conglycinin by altering protein conformation. Meinlschmidt et al. [59] demonstrated that high-pressure (higher than 300 MPa) assisted enzymatic hydrolysis may be used in the production of tasty soy-based food ingredients to reduce Gly m5 immunoreactivity. However, the protein matrix (i.e., the seed structure) plays a significant role in protein stability and may influence processing outcomes [60]. In our previous work on commercially available soy isolates, concentrates, and hydrolysates [34], we observed lower numbers of fractions reactive with sera from soy-allergic patients. Nevertheless, some immunoreactive fractions, corresponding to molecular weights of approximately 39, 45, and 55 kDa, were still detectable even in hydrolysed commercial preparations. In the present study, similar immunoreactive fractions were identified in our experimental 7S(i) and 11S(i) isolates, indicating that these proteins retained their immunoreactivity. These observations align with reports showing that thermal processing may not always decrease allergenicity. For instance, recent findings demonstrated that heating SPI at 60–100 °C for 20 min increased its IgE-binding capacity by 13.1–31.6%, accompanied by protein unfolding, aggregate formation, and exposure of conformational epitopes. LC–MS/MS analyses further indicated that heating can mask or reveal linear epitopes in major allergens (Gly m 4, Gly m 5, Gly m 6, P28, Kunitz trypsin inhibitor), thereby enhancing immunoreactivity [61,62]. Although in our study thermal treatments (particularly pressure cooking) generally reduced IgE reactivity of 7S and 11S, the persistence of specific immunoreactive fractions suggests that structural modifications induced by heat may either diminish or, under certain conditions, enhance epitope accessibility. This highlights the complexity of thermal effects on soy allergens and reinforces the importance of evaluating both degradation and potential neoepitope formation when assessing the safety of processed soy proteins.

Gan et al. [63] reported that phenolic acids present in soybeans form stable complexes with proteins, affecting their digestibility. The strength of these interactions depends on the structure of both the phenolic compound and the specific protein fraction involved. Our results support this concept, as significant changes in immunoreactivity were only observed in isolated 7S(i) and 11S(i) fractions subjected to thermal processing. The extent of these changes depended on the type of cooking method, the duration of the treatment, and possible interactions with remaining components in the isolates, such as residual phenolics, carbohydrates, or lipids. Moreover, the extent of these changes depended on the cooking method, duration, and other physicochemical parameters. Among the methods tested, pressure-cooking was the most effective in reducing immunoreactivity.

This intensive treatment, involving simultaneous exposure to high pressure and temperature, proved effective even for the 7S(i) fraction. Cabanillas et al. [64] previously noted the high stability of this fraction, which retains its allergenic potential during pressure-cooking due to its compact structure and disulfide bonds stabilizing conformational epitopes. Nevertheless, as shown in our study, the simultaneous application of high temperature and pressure (i.e., pressure-cooking) to the isolated 7S(i) fraction, devoid of its protective matrix, reduced its immunoreactivity by up to 71%. It should be noted, however, that our study was conducted on isolated protein fractions rather than whole soybean seeds or complete food matrices. This isolation removes the protective effects of the protein matrix and other seed components, which may stabilize proteins and limit their denaturation during thermal treatment. As a result, the effects observed in isolated proteins may differ from those occurring in seeds, where interactions between proteins, carbohydrates, lipids, polyphenols, and other ingredients can influence both protein structure and epitope accessibility.

Culinary treatments may then have a significant influence on immunoreactivity and, consequently, allergenicity of foodstuffs. However, their final impact is influenced by numerous factors, making it difficult to draw definitive conclusions without further rigorous investigation. As Poms & Anklam [65] noted, microwave heating and heating to 100 °C reduce allergenicity of soybeans but do not eliminate adverse reactions in highly sensitive individuals. To accurately assess the allergenic potential of treated fractions, the patient’s serum should be used.

Although soy has been traditionally classified as a priority allergen, recent evaluations have questioned the global relevance of its allergenic potential. According to the FAO/WHO Expert Committee [66], the inclusion of soy as a priority allergen under the Codex Alimentarius is controversial. Based on current evidence regarding its relatively low prevalence and potency, the committee recommended the removal of soy from the list of global priority allergens. Supporting this reassessment, Turner et al. [67] identified only five relevant studies in a comprehensive literature review, and no cases of anaphylaxis were reported at low exposure levels (<200 mg protein). These findings are consistent with data from Conrado et al. [68], which indicate that soybean is an uncommon cause of anaphylaxis globally.

Considering the observed reduction in immunoreactivity of soy proteins through culinary processing, particularly pressure cooking, may be especially relevant for consumers with mild or moderate soy allergy. Nonetheless, despite this favorable toxicological profile and decreasing global prioritization, soy may still pose a risk to highly sensitized individuals. Therefore, efforts to reduce immunoreactivity remain justified, particularly in the context of food labeling and hypoallergenic food development.

Authors should discuss the results and how they can be interpreted from the perspective of previous studies and of the working hypotheses. The findings and their implications should be discussed in the broadest context possible. Future research directions may also be highlighted.

## 5. Conclusions

Culinary treatments of the 7S(i) and 11S(i) protein fractions, performed outside the protective seed matrix, significantly reduce their immunoreactivity, suggesting a potential decrease in allergenic potential, which was observed using polyclonal antibodies. Pressure cooking was the most effective method, reducing immunoreactivity by 71% and 58% for 7S(i) and 11S(i), respectively.

These findings indicate that such a reduction may be sufficient to lower the risk of allergic reactions in individuals with mild soy allergy. However, the persistence of immunoreactive bands even after intensive treatment suggests that culinary methods alone are unlikely to ensure safety for highly sensitized individuals.

Further research is warranted to explore strategies for minimizing soy protein immunoreactivity and to assess the clinical relevance of such modifications using patient sera—particularly in the context of hypoallergenic product development and accurate food labeling. It is also important to consider that different soybean varieties may respond differently to processing, so future studies should examine more varieties.

The methodology used does not fully simulate the complex immunological reactions occurring in vitro; therefore, further studies should include more complex models of immunological reactivity using animal models. This would allow for the reflection of a more dynamic and multifaceted immune response to the tested proteins.

## Figures and Tables

**Table 1:** Basic composition of soybeans and the 7S and 11S protein fractions obtained from seeds and seed after different culinary treatments (letters indicate statistical comparisons within each fraction).

Analyzed Material	Protein [%d.wt.]	Ash [%d.wt.]	Fat [% d.wt.]
Soybeans	43.30 ± 0.82	5.67 ± 2.46	20.03 ± 0.8
7S(i)	98.47 ± 1.31 ^a^	1.40 ± 1.00 ^a^	0.00
11S(i)	96.14 ± 1.40 ^A^	3.61 ± 1.82 ^A^	0.00
7S(sb60)	97.92 ± 1.32 ^a^	1.98 ± 0.89 ^a^	0.00
11S(sb60)	96.06 ± 1.24 ^A^	3.25 ± 1.23 ^A^	0.00
7S(sb120)	98.02 ± 1.16 ^a^	1.38 ± 0.78 ^a^	0.00
11S(sb120)	96.60 ± 1.32 ^A^	2.98 ± 1.13 ^A^	0.00
7S(smv)	98.12 ± 1.74 ^a^	1.62 ± 0.91 ^a^	0.00
11S(smv)	96.29 ± 1.51 ^A^	3.05 ± 1.08 ^A^	0.00
7S(spc)	98.28 ± 1.29 ^a^	1.61 ± 1.03 ^a^	0.00
11S(spc)	96.17 ± 1.52 ^A^	3.45 ± 1.27 ^A^	0.00

^a,A^—values within the same column followed by the same letter are not significantly different (*p* > 0.05).

**Table 2:** Relative content (%) of immunoreactive 7S(i) and 11S(i) protein fractions isolated from soybean seeds and subjected to different cooking methods determined by ELISA.

Fractions	Culinary Treatment	Remaining Immunoreactive Protein Content [%]
7S(i)	Untreated	100.00
	Boiling (100 °C/60 min)	73.62 ± 2.87 ^c^
	Boiling (100 °C/120 min)	46.15 ± 2.25 ^b^
	Pressure cooker (202 kPa/50 min, 121 °C)	28.54 ± 1.99 ^a^
	Microwave oven (360 W/30 min)	48.15 ± 2.41 ^b^
11S(i)	Untreated	100.00
	Boiling (100 °C/60 min)	61.91 ± 2.35 ^C^
	Boiling (100 °C/120 min)	34.91 ± 2.44 ^A^
	Pressure cooker (202 kPa/50 min, 121 °C)	42.14 ± 1.99 ^B^
	Microwave oven (360 W/30 min)	61.23 ± 2.67 ^C^

^a,b,c,A,B,C^—different letters indicate significant differences (*p* ≤ 0.05).

## Data Availability

The original contributions presented in the study are included in the article; further inquiries can be directed to the corresponding author.
